# Expression stability of commonly used reference genes in canine articular connective tissues

**DOI:** 10.1186/1746-6148-3-7

**Published:** 2007-05-07

**Authors:** Duncan Ayers, Dylan N Clements, Fiona Salway, Philip JR Day

**Affiliations:** 1 Centre for Integrated Genomic Medical Research, The Manchester Interdisciplinary Biocentre, University of Manchester, M1 7ND, UK; 2 Musculoskeletal Diseases Research Group, Faculty of Veterinary Science, University of Liverpool. UK; 3 ISAS – Institute for Analytical Sciences, Bunsen-Kirchhoff-Str. 11, 44139 Dortmund, Germany

## Abstract

**Background:**

The quantification of gene expression in tissue samples requires the use of reference genes to normalise transcript numbers between different samples. Reference gene stability may vary between different tissues, and between the same tissue in different disease states. We evaluated the stability of 9 reference genes commonly used in human gene expression studies. Real-time reverse transcription PCR and a mathematical algorithm were used to establish which reference genes were most stably expressed in normal and diseased canine articular tissues and two canine cell lines stimulated with lipolysaccaride (LPS).

**Results:**

The optimal reference genes for comparing gene expression data between normal and diseased infrapatella fat pad were *RPL13A *and *YWHAZ *(M = 0.56). The ideal reference genes for comparing normal and osteoarthritic (OA) cartilage were *RPL13A *and *SDHA *(M = 0.57). The best reference genes for comparing normal and ruptured canine cranial cruciate ligament were *B2M *and *TBP *(M = 0.59). The best reference genes for normalising gene expression data from normal and LPS stimulated cell lines were *SDHA *and *YWHAZ *(K6) or *SDHA *and *HMBS *(DH82), which had expression stability (M) values of 0.05 (K6) and 0.07 (DH82) respectively. The number of reference genes required to reduce pairwise variation (V) to <0.20 was 4 for cell lines, 5 for cartilage, 7 for cranial cruciate ligament and 8 for fat tissue. Reference gene stability was not related to the level of gene expression.

**Conclusion:**

The reference genes demonstrating the most stable expression within each different canine articular tissue were identified, but no single reference gene was identified as having stable expression in all different tissue types. This study underlines the necessity to select reference genes on the basis of tissue and disease specific expression profile evaluation and highlights the requirement for the identification of new reference genes with greater expression stability for use in canine articular tissue gene expression studies.

## Background

Quantification of gene expression in diseased tissue can determine genes which are involved in the development or progression of disease [1], suitable for genomic evaluation [2] or gene based prognostics [3] or treatment [4]. Real-time reverse transcription polymerase chain reaction (real-time RT-PCR) is the most commonly utilised method of measuring gene expression in biological systems. The method provides accurate quantification of transcript number, good sensitivity over a wide range of transcript expression levels, and increasing high throughput capabilities. Several factors contribute to errors of variation in gene expression measurement, including issues relating to sample starting cell number and sample cell types, mRNA extraction protocol and handling techniques [5], mRNA quality [6,7], method of reverse transcription [8] and analytical detection chemistry method [5].

To accommodate these differences in RNA sample preparation and analysis, the measurement of relative expression of transcript has evolved a means to control these variables employing a process that is termed normalisation [9]. Normalisation of real-time RT-PCR data is classically performed through the selection of a calibrant internal control gene, known as a reference gene or "house-keeping" gene. Conceptually, an ideal gene selected as an internal reference control should have a constant level of expression across the tissue or cell samples used throughout the experiment, and should not exhibit altered expression with diseased or, control tissues, or indeed experimental conditions [10]. Initially, ubiquitously applied reference genes were sought that could be applied across tissue and experimental types [3]. However, recent studies have shown that the expression stability of some of the commonly used reference genes, such as *B2M*, *GAPDH *and *ACTB *is not constant for all tissues or disease states [10,11].

Current studies identify reference genes that are validated for each tissue or cell type and disease or experiment. These reference genes can be selected by evaluating data from real-time RT-PCR statistical algorithms, such geNorm [11], Global Pattern Recognition [12], Bestkeeper [13], Normfinder [14] or equivalence tests [15]. The principal of the geNorm algorithm is that from an initial group of candidate reference genes tested across all the types of tissue studied and the experimental conditions, the expression ratio of the two reference genes that display the most similar expression identified these genes as the best choice to monitor variation in test gene expression [11]. Global Pattern Recognition is a statistical algorithm which compares the expression or each gene to every other gene used in the comparison, similar to analysis of variance (ANOVA) but with exclusion of nonsensical data (e.g. threshold cycle (C_T_) values of 40, where no amplification has taken place) [12]. The Bestkeeper algorithm measures the geometric mean of reference gene crossing point values, to determine the optimal reference gene for use in a samples set. [13]. Equivalence testing is the mathematical determination of the standard deviation of differences in expression values between samples being compared [15]. The Normfinder algorithm uses a model-based approach to the estimation of expression variation, which takes into account variation across sub-groups and avoids the artificial selection of co-regulated genes [14].

Osteoarthritis (OA) is a condition characterised by the destruction of articular cartilage, resulting in pain and dysfunction of the affected joint. OA is a prevalent disease of mammalian joints, which affects up to 20% of the canine population at large [16] with the hip, stifle or elbow joints most commonly affected. The prevalence of OA on radiographic evaluation of dog populations is much higher, with estimates of up to 73% of individuals in a single breed having radiographic evidence of OA of the hip, or a disease (hip dysplasia) which can lead to OA. [17]. OA can be experimentally induced in canine joints through surgical procedures, such as cranial cruciate ligament transaction [18], and OA associated with naturally occurring cranial cruciate ligament rupture is identified in dogs [19].

To date, the majority of molecular research into OA has investigated the mechanisms involved in the catabolism of articular cartilage. However, OA is not solely a disease of articular cartilage, as there are changes in other articular tissues, such as fat [20] and ligaments [21]. The quantification of gene expression in all articular connective tissues, such as cartilage [22], cranial cruciate ligament [23], and infrapatella fat [20] will help to determine the molecular pathogenesis of OA.

In this paper, we identify the best reference genes for use in real-time RT-PCR experiments investigating gene expression in canine articular connective tissue studies. The study draws upon reference genes used in studies evaluating dys-regulation of gene expression in human tissue [11], and employs them to determine if these have similar application in canine studies. The geNorm algorithm is employed to investigate the expression stability of 9 commonly used reference genes (glyceraldehyde-3-phosphate dehydrogenase [*GAPDH*], beta-actin [*ACTB*], beta-2-microglobulin [*B2M*] hydroxymethylbilane synthase [*HMBS*], hypoxanthine guanine phosphoribosyl transferase [*HPRT*], ribosomal protein L13a [*RPL13A*], succinate dehydrogenase flavoprotein subunit A [*SDHA*], TATA box binding protein [*TBP*] and tyrosine 3-monooxygenase/tryptophan 5-monooxygenase activation protein, zeta polypeptide [*YWHAZ*]) [11] in normal and diseased canine connective tissues. We hypothesised that some of the genes selected would demonstrate stable expression in the different canine connective tissues investigated and could therefore be applied as reference genes to normalise future real-time RT-PCR studies that evaluate gene expression in canine on connective tissues.

## Results

**Figure 1:**
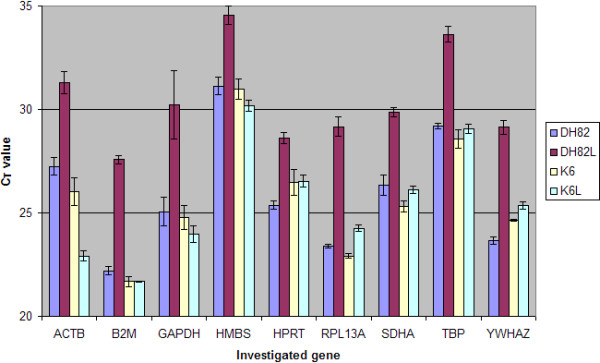
Mean expression values (± Standard Deviation [SD]) of canine cell lines (DH82, K6) before and after lipopolysaccharide stimulation (DH82L, K6L), following RT-PCR analysis.

**Figure 2:**
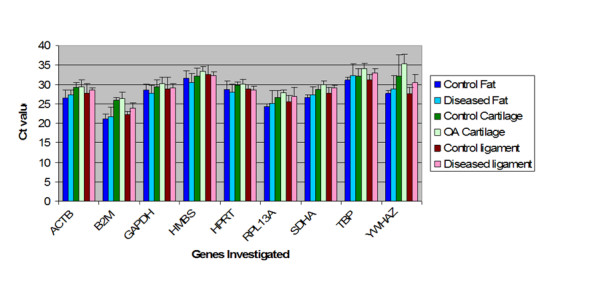
Mean expression values for candidate reference genes (± SD) from healthy and diseased canine infrapatellar fat tissue, canine cartilage and ligament tissue, following RT-PCR analysis.

The gene expression levels (threshold cycle, C_T_) for each sample group were averaged with standard deviation was plotted for each group of samples (Figures 1 and 2).

The results for each of the stimulated canine cell lines demonstrated that optimum reference genes had an M value of 0.05 (K6; *SDHA *and *YWHAZ*) to 0.07 (DH82; *SDHA *and *HMBS*) (Table 1). However, when examining both cell lines simultaneously, the recommended reference genes were *GAPDH *and *B2M*, and the M value was increased (0.34, Table 1), indicating reduced stability. Reference gene expression levels in the K6 cell line appeared to be higher when compared to DH82 cell lines, with most of the K6 C_T _values being less than 30 (Figure 1).

The genes with the highest expression stability for normal fat pad joints were *RPL13A *and *TBP *(M = 0.56), which differed to OA fat pad (*GAPDH *and *HPRT*, M = 0.34) [Table 2]. The expression stability of the ideal reference genes for use in studies comparing both normal and OA fat pad (*RPL13A *and *YWHAZ*) when analysed together had an M value of 0.55. The C_T _results showed a relatively consistent level of expression for each gene analysed in both sample groups (Figure 2). One gene (*ACTB*) was consistently one of the two least stably expressed genes in both normal and OA fat and when comparing both groups of tissue.

The optimal reference genes for use in canine articular cartilage tissue samples were *ACTB *and *SDHA *(M = 0.47) for normal cartilage, and *HPRT *and *TBP *for OA (M = 0.45) articular cartilage (Table 3). When both sample groups were analysed together, the expression stability was reduced (M value = 0.57, Table 3), with *SDHA *and *RPL13A *identified as the most consistently expressed reference genes. The expression levels of each investigated gene in the OA sample group did not demonstrate marked variation in expression when compared to the C_T _values of healthy cartilage (Figure 2). One gene (*YWHAZ*) was the least stably expressed in both normal and OA cartilage and when comparing both groups of tissue.

The expression stability of the optimal reference genes for normal cranial cruciate ligament tissue (*SDHA *and *YWHAZ*, M value = 0.26) and diseased tissue (*SDHA *and *HPRT*, M value = 0.33) (see Table 4) were broadly similar. Analysis of the sample groups together identified reduced expression stability (0.59) of the two optimal reference genes (*TBP *and *B2M*). The CT results demonstrated a notable decrease in gene expression for B2M and YWHAZ from the OA sample group (Figure 2).

**Figure 3:**
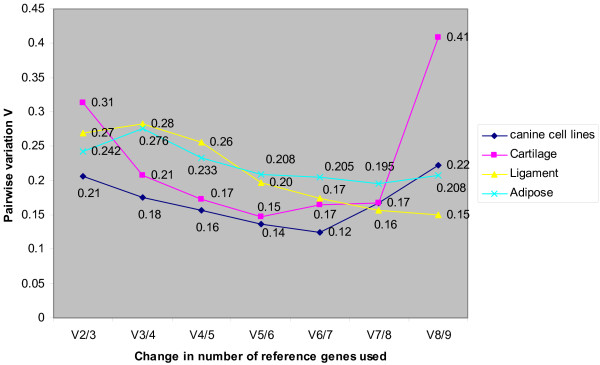
Pairwise variation analysis for determining optimal number of reference genes in various canine tissues (healthy and diseased).

The ideal number of reference genes required to reduce pairwise variation (V) to <0.20 was 4 for canine cell lines, 5 for cartilage, 7 for cranial cruciate ligament and 8 for fat tissue (Figure 3). Addition of further reference genes below the threshold V value of 0.2 did not greatly reduce pairwise variation any further. The increase in V values observed following the hypothetical utilisation of a total of eight reference genes (canine cell lines) or nine genes (fat and cartilage) was consistent with previous reports [11], and has been ascribed to the additional reference gene(s) having the worst expression stability characteristics [11].

A strong correlation between transcript quantity and reference gene stability was only identified for one experiment (LPS stimulation of DH82 cells, R = -0.617). All other experiments where determined to have low correlation between reference gene stability and transcript quantity (r range = 0.238 to -0.280).

## Discussion

At present there is no consensus as to which stability algorithm should be used to optimise reference gene stability. The model based approach is the best for analysing genes whose function is poorly defined (such as new potential genes from microarray data), and therefore may have the potential to be co-regulated as pairwise measurements of stability will artificially tend to select co-regulated genes [14]. Comparisons of the different methods of candidate reference gene selection tend to identify the best and the worst reference genes consistently [24-26] when evaluating reference genes whose functions are well defined. As there is no "gold standard" technique for determining the rank orders of candidate reference genes. We used the geNorm algorithm because in provided both measures of individual gene stability, and the measures of pairwise variation for groups of genes. Furthermore accepted threshold measures of gene stability and pairwise variation are well described with this method [11], and the genes we evaluated have well characterised biological functions, and for whom no co-regulation has previously been reported.

To date, limited information has been published on the selection of appropriate reference genes for use in the quantification of gene expression in mammalian articular connective tissues or canine tissues in general. Reports describing the optimisation of reference genes for use in canine mammary tumours are published [27], as well as those for use in prostate, kidney, mammary gland, and left ventricle of five to nine dogs with or without undefined diseases [28]. We investigated the stability of reference genes in a number of different tissues affected by disease (i.e. tissues from which meaningful comparisons of gene expression would be made), and therefore for which the comparison and optimisation of different reference genes is important.

The expression levels of different reference genes (elongation factor 1-alpha, *GAPDH*, actin) have been compared in human articular cartilage using a crudely quantitative method, and displayed that the expression level of each reference gene was raised in OA articular cartilage [29]. To our knowledge, only one of the reference genes (*B2M*) we evaluated to be consistently expressed has been reported as being differentially expressed in OA articular cartilage [30]. Yet despite the lack of information on suitable reference gene selection, the majority of studies quantifying gene expression in connective tissues use *GAPDH *as the reference gene. *GAPDH *did not demonstrate stable expression across all tissues in this study, or in previous studies of reference gene stability in different canine tissues [27,28]. Indeed, no single reference gene was identified as having stable expression when analysing the data from normal and diseased tissues separately and combined, which is also consistent with previous reports evaluating canine [28] and human [11] tissue.

The relationship between the stability value of a reference gene and the C_T _value of that gene was measured, as genes which are less abundantly expressed will be more susceptible to errors in measurement due to small variations from the calculated efficiency values. However, we found the stability of individual reference genes (M Value) appears to be unrelated to the level of expression (C_T_) which was consistent with a previous report [28], with the exception of one experiment which was probably a type II statistical error.

The justification of using the selected genes as reference genes in canine tissue is based on published work defining their stability as for use as reference genes with human tissue [11,31], or canine tissue [28]. The function of these genes in canine cells is assumed to be the same as that reported for humans. The expression stability of the two sets of reference genes within the canine cell lines was evaluated, using LPS stimulation to mimic biological variation seen between normal and diseased tissue. Interestingly, the expression stability of the reference genes in the cell lines was much higher than those reported with tissue samples, (lower M values shown in Table 1) which reflects the benefits of working with cell cultures compared to clinical tissue specified. Cell cultures should allow more control over the heterogeneity of cell type, sample handling, storage and mRNA recovery, thereby minimising the level of degradation frequently identified with pathological clinical tissue [32], which may in turn affected gene expression measures [6].

A pair wise variation (V) of 0.15 has been recommended as an arbitrary cut-off point below which the inclusion of additional reference genes expression was not required [11], although this degree of reference gene expression stability could not be achieved for fat tissue or cranial cruciate ligament. If the selected V value was increased to 0.2, then pairwise stability could be achieved using between 4 genes (cell lines) and 8 genes (fat tissue). Alternative measures of disease status, such as histological grading [33] to further select tissue specimens in comparable stages of disease may have helped reduce the variability in reference gene stability, and thus the number of reference genes required for each experiment.

The selection of candidate genes based on ontological function, combined with the evaluation gene expression microarray experiments [25] may reduce the number of reference gene required to obtain acceptable expression stability between samples, as clearly the number of genes suggested are too large to be used in practice. However, until these have been identified, this experiment gives an indication of which genes can be used for the normalisation of gene expression measures in canine OA tissues, and which genes are not suitable for normalising real-time RT-PCR data from fat (*ACTB*) or cartilage (*YWHAZ*). Ultimately, we found that reference gene optimisation has to be performed on a tissue and disease specific basis, even when evaluating tissue from the same or similar diseases.

## Conclusion

No single reference gene was identified as having stable expression in all canine articular tissue types. The combinations of reference genes required to demonstrate stable expression in each tissue were also identified. The number of genes required to achieve stability for comparing normal and diseased infrapatella fat and cranial cruciate ligament were inappropriate for routine application. This study underlines the necessity to select reference genes on the basis of tissue and disease specific expression evaluation and highlights the requirement for the identification of new reference genes with greater expression stability for use in canine articular tissue gene expression studies.

## Methods

### Cell Line Culture

Canine K6 and DH82 canine cell lines were used in this study. Cells from the K6 cell line were myelomonocytes [34], whilst cells derived from the DH82 cell line were of macrophage-monocytic origin [35]. These cell lines were cultured and harvested as previously described [36]. Twenty-four hour lipopolysaccharide (LPS) stimulation of both cell lines was performed by the addition of 100 μg/mL LPS (*Salmonella typhimurium*; Sigma, Poole, UK) to the media. Details of cell culture treatments and procedures are available in reference [36]. Cells were harvested after washing with fresh media by repeated passage of 1 ml of phenol/guanidine HCl reagent (Trizol™; Invitrogen, Dorset, UK). Total RNA was extracted and isolated using spin columns (RNeasy; Qiagen Ltd, Crawley, UK) as described by Reno *et al *[37].

### Tissue Samples

Articular cartilage, cranial cruciate ligament and infrapatella fat samples were obtained from healthy and diseased dogs. Infrapatella fat samples (n = 5) were obtained from dogs with clinical OA (secondary to naturally occurring joint disease). Osteoarthritic articular cartilage samples (n = 5) were obtained from dogs undergoing total hip replacement, and ruptured cranial cruciate ligament samples (n = 5) were obtained from dogs undergoing a routine surgical procedure (exploratory arthrotomy) for the treatment of the naturally occurring joint disease, which radiographic and macroscopic evidence of osteoarthritis. Control samples (healthy) were obtained from the stifles (infrapatella fat (n = 5) and cranial cruciate ligament (n = 5) and hips (articular cartilage (n = 5) of dogs of normal bodyweight euthanized for reasons other than, and with no evidence of, joint disease. All samples were stored in RNAlater™ (Ambion Ltd; Huntingdon, UK) at room temperature immediately after harvesting and maintained at -20°C after 24 hours until use.

### Total RNA Extraction

The tissue samples were removed from RNAlater™ and total RNA was extracted using phenol/guanidine HCl reagents. For (i) cranial cruciate ligament and articular cartilage, and (ii) articular fat pad, Trizol™ (Invitrogen Ltd) and Qiasol (Qiagen Ltd, Crawley, UK) were used respectively, employing the standard instructions as recommended by the manufacturers.

An on column DNA digestion step was included (RNase-Free DNase Set; Qiagen Ltd). Final elution of the total RNA was performed using 30 μl of RNase free water, and repeated to maximise the amount of RNA eluted. Total RNA samples were stored at -80°C until use. The concentration of total RNA representing each sample was quantified by using a NanoDrop ND1 spectrophotometer (NanoDrop Technologies Ltd, Utah, USA). RNA integrity was analysed by evaluating the capillary electrophoresis trace (Agilent Bioanalyser 2100; Agilent Technology, California, USA) of each sample using the RNA integrity number [RIN] algorithm [38] and Degradation Factor [DF] [39]. From these quality control calculations, all RNA samples were determined to have no, or mild, loss of integrity (RIN > 6.5, and/or DF < 8), and thus deemed suitable for use in the following experiments [32].

### PCR Assay Design

**Figure 4:**
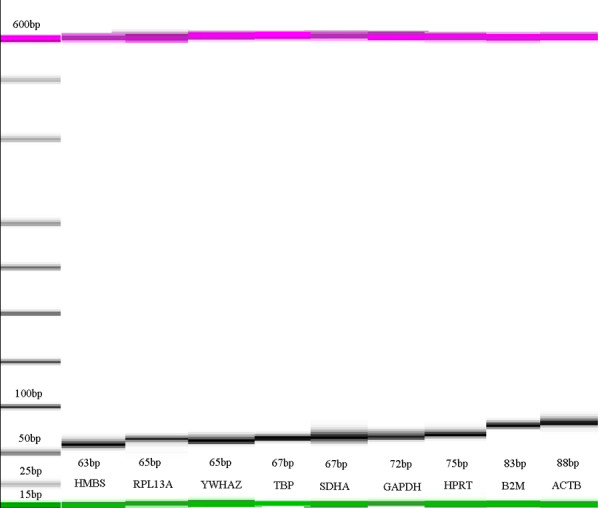
Microfluidic capillary electrophoresis of the sequence reactions identified single bands which corresponded exactly to the expected product size.

Primer and probe sequences were designed for nine of the most commonly used control (reference) genes in man [11] using the Universal Probe Library Assay Design Centre [40]. Transcript sequences were obtained from the canine genome database [41], with cross reference to the National Centre for Biotechnology Information [42]. Primers and matched probes were selected for; glyceraldehyde-3-phosphate dehydrogenase [*GAPDH*], beta-actin [*ACTB*], beta-2-microglobulin [*B2M*] hydroxymethylbilane synthase [*HMBS*], hypoxanthine guanine phosphoribosyl transferase [*HPRT*], ribosomal protein L13a [*RPL13A*], succinate dehydrogenase flavoprotein subunit A [*SDHA*], TATA box binding protein [*TBP*] and tyrosine 3-monooxygenase/tryptophan 5-monooxygenase activation protein, zeta polypeptide [*YWHAZ*] (Table 5). Basic Local Alignment Search Tool [42] searches were performed for all primer sequences to confirm gene specificity. To enhance the probability of transcript-specific PCR, selected amplicon systems were designed so that the last six to seven bases of a 3' primer or the probe crossed an exon-exon boundary. When this was not possible, the primers were designed to be hybridised on different exons, with an intronic sequence greater than 1,100 base pairs, to maintain specificity for mRNA. Some assays could be designed within only a single exon, and thus a genomic DNA assay [43] was also used to determine whether genomic contamination was present. No genomic DNA was identified in any sample. The specificity of primer sets was further confirmed through microfluidic capillary electrophoresis (using the Agilent 2100 Bioanalyzer, Agilent Technologies UK Ltd, South Queensferry, West Lothian, UK) to measurement quantify the size of any product(s) generated by the real-time RT-PCR reaction. For each probe and primer set a single band of the expected size was identified (Figure 4). 5' reporter dye FAM (6-carboxy fluorescein) and dark quencher dye probes were synthesized by Exiqon (Vedbaek, Denmark), and primers were synthesized by Metabion International AG (Martinsried, Germany).

### Reverse Transcription

Reverse transcription was performed using 200 ug total RNA with oligo-dT and Superscript II reverse transcriptase (Invitrogen). The real-time PCR assays were all performed in triplicate using a TaqMan™ ABI PRISM 7700 SDS (Applied Biosystems, California, USA) employing 96-well plates, and no template controls were used for each assay. Each assay well had a 20 μL reaction volume consisting of 10 μL 2 × PCR master mix with uracil N-glycosylase (Applied Biosystems), 3.8 μL of sterile distilled water, and 0.4 μL each of 20 μM forward and reverse primers, 0.4 μL of 20 μM probe (Exiqon, Vedbaek, Denmark) and 5 μL of sample cDNA (templates) or water (negative controls). The amplification was performed according to the standard protocol with 40 cycles of 95°C for 15 sec and 60°C for 1 min as recommended by the manufacturer (Applied Biosystems). Real-time data was analyzed by using the Sequence Detection Systems software, version 1.6.3 (Applied Biosystems). Standard curves were generated from five repeated ten-fold serial dilutions of cDNA.

### Data Analysis

Following the RT-PCR assays, the C_T _values for each of the candidate reference genes were converted into relative quantities using the algorithm described by Vandesompele et al [11]. These relative quantities were then entered into a data input file which could be analyzed by the geNorm software package to identify which of the assayed genes for a given tissue type exhibited the most stable relative expression. Gene expression stability measures (M value) of each individual gene within the tissue evaluated was calculated by the geNorm software. The pairwise variation (V Value), which is an indication of the influence on the stability attributed by addition of a gene to a group of reference genes, was also calculated by the geNorm software package. Genes with the lowest M value are the most stably expressed. Pairwise variation between samples is (usually) reduced by the inclusion of additional reference genes thus it is an indication of the number of genes required to achieve and arbitrarily selected measure of reference gene stability. To determine the effect of expression level on gene expression stability, correlations between the level of expression (C_T _value) and reference gene stability (M value) were performed using a Spearmans rank correlation coefficient (Minitab 14.0, State College PA, USA).

Spearman rank correlation coefficients were calculated for each experiment to determine the interaction between transcript quantity (C_T _value) and reference gene stability (rank order, as determined in tables 1, 2, 3, 4).

## Abbreviations

*GAPDH *= glyceraldehyde-3-phosphate dehydrogenase, *ACTB = *beta-actin, *B2M *= beta-2-microglobulin, *HMBS *= hydroxymethylbilane synthase, *HPRT *= hypoxanthine guanine phosphoribosyl transferase, *RPL13A *= ribosomal protein L13a, *SDHA *= succinate dehydrogenase flavoprotein subunit A, *TBP *= TATA box binding protein and *YWHAZ *= tyrosine 3-monooxygenase/tryptophan 5-monooxygenase activation protein, zeta polypeptide, C_T _= Threshold cycle, ANOVA = Analysis of variance, LPS = Lipopolysaccaride, V = Pairwise variation, M = Gene Stability Value, RIN = RNA integrity number, DF = Degradation Factor, RT-PCR = reverse transcription polymerase chain reaction, OA = Osteoarthritis

## Authors' contributions

DA and FS carried out the assay design, the molecular genetic studies and performed the statistical analysis. DNC collected and processed samples, and performed some of the statistical analysis. DNC and PJRD conceived the study, its design and coordination, and drafted the manuscript. All authors read and approved the final abstract. DA was self funded, DNC was funded by the Biotechnology and Biological Sciences Research Council, FS was funded by the University of Manchester, and PJD was funded by the Higher Education Funding Council of England. The study was funded in part by a grant from the PetPlan charitable trust, UK, and in part by a project grant from the University of Manchester. The manuscript preparation was funded by the University of Manchester. Neither funding body had any role in the study design; in the collection, analysis, and interpretation of data; in the writing of the manuscript; or the decision to submit the manuscript for publication.

## Figures and Tables

**Table 1:** Canine cell lines (stimulated with LPS) reference genes ranked in order of increasing expression stability (Increases from top to bottom).

**Number of remaining reference genes**	**Combined Cell lines**	**M Value**	**V Value**	**DH82 Cells**	**M Value**	**V Value**	**K6 Cells**	**M Value**	**V Value**
9	*ACTB*	1.24	0.222	*RPL13A*	0.83	0.114	*ACTB*	1.03	0.263
8	*HPRT*	1.00	0.167	*YWHAZ*	0.78	0.115	*RPL13A*	0.64	0.113
7	*YWHAZ*	0.85	0.125	*B2M*	0.73	0.142	*GAPDH*	0.56	0.102
6	*HMBS*	0.79	0.137	*GAPDH*	0.62	0.169	*HMBS*	0.50	0.142
5	*SDHA*	0.73	0.156	*TBP*	0.43	0.126	*B2M*	0.32	0.073
4	*RPL13A*	0.65	0.175	*ACTB*	0.29	0.112	*HPRT*	0.29	0.109
3	*TBP*	0.54	0.207	*HPRT*	0.14	0.059	*TBP*	0.14	0.063
**2 Most stably expressed**	***B2M/GAPDH***	**0.34**		***HMBS/SDHA***	**0.07**		***SDHA/YWHAZ***	**0.05**	

**Table 2:** Canine infrapatella fat tissue reference genes ranked in order of increasing expression stability (Increases from top to bottom).

**Combined**	**M Value**	**V Value**	**Normal fat pad**	**M Value**	**V Value**	**OA Fat pad**	**M Value**	**V Value**
*ACTB*	1.57	0.21	*ACTB*	1.49	0.23	*SDHA*	1.26	0.15
*HMBS*	1.43	0.20	*HPRT*	1.29	0.21	*ACTB*	1.17	0.18
*HPRT*	1.31	0.205	*HMBS*	1.13	0.20	*YWHAZ*	1.05	0.14
*GAPDH*	1.20	0.208	*GAPDH*	0.98	0.17	*RPL13A*	1.01	0.18
*SDHA*	1.08	0.23	*B2M*	0.88	0.23	*TBP*	0.92	0.19
*B2M*	0.94	0.28	*SDHA*	0.65	0.16	*B2M*	0.82	0.21
*TBP*	0.70	0.24	*YWHAZ*	0.57	0.17	*HMBS*	0.70	0.29
***RPL13A/YWHAZ***	**0.55**		***RPL13A/TBP***	**0.56**		***GAPDH/HPRT***	**0.34**	

**Table 3:** Canine cartilage tissue reference genes ranked in order of increasing expression stability (Increases from top to bottom).

**Combined**	**M Value**	**V Value**	**Healthy Cartilage**	**M Value**	**V Value**	**OA cartilage**	**M Value**	**V Value**
*YWHAZ*	1.75	0.41	*YWHAZ*	1.88	0.51	*YWHAZ*	1.55	0.31
*B2M*	1.18	0.17	*B2M*	1.10	0.15	*B2M*	1.17	0.18
*HMBS*	1.08	0.17	*HPRT*	1.02	0.17	*HMBS*	1.03	0.20
*HPRT*	0.99	0.15	*GAPDH*	0.90	0.14	*ACTB*	0.85	0.15
*ACTB*	0.95	0.17	*TBP*	0.86	0.16	*RPL13A*	0.77	0.17
*GAPDH*	0.90	0.21	*HMBS*	0.79	0.20	*SDHA*	0.68	0.19
*TBP*	0.84	0.31	*RPL13A*	0.70	0.26	*GAPDH*	0.53	0.18
***RPL13A/SDHA***	**0.57**		***ACTB/SDHA***	**0.47**		***HPRT/TBP***	**0.45**	

**Table 4:** Canine ligament tissue reference genes ranked in order of increasing expression stability (Increases from top to bottom).

**Combined**	**M Value**	**V Value**	**Healthy Ligament**	**M Value**	**V Value**	**Ruptured Ligament**	**M Value**	**V Value**
*YWHAZ*	1.39	0.15	*GAPDH*	1.28	0.16	*RPL13A*	1.10	0.17
*RPL13A*	1.33	0.16	*ACTB*	1.17	0.19	*YWHAZ*	0.94	0.16
*GAPDH*	1.28	0.17	*HMBS*	1.02	0.25	*B2M*	0.80	0.15
*HMBS*	1.22	0.20	*B2M*	0.71	0.15	*GAPDH*	0.69	0.14
*ACTB*	1.15	0.26	*HPRT*	0.60	0.17	*HMBS*	0.59	0.11
*HPRT*	0.98	0.28	*TBP*	0.41	0.12	*TBP*	0.57	0.15
*SDHA*	0.75	0.27	*RPL13A*	0.31	0.10	*ACTB*	0.48	0.17
***B2M/TBP***	**0.59**		***SDHA/YWHAZ***	**0.26**		***HPRT/SDHA***	**0.33**	

**Table 5:** Sequences for primers and probes for candidate reference genes used in the study (5'-3' direction).

**Gene**	**Forward Primer**	**Reverse Primer**	**Probe**	**Amplicon Length Bp**	**Efficiency of Assay (%age)**	**Accession Number for sequence used**
*GAPDH*	CTGGGGCTCACTTGAAAGG	CAAACATGGGGGCATCAG	CTGCTCCT	72	102.3	NM_001003142
*ACTB*	CCCATCTACGAGGGGTACG	GGATCTTCATGAGGTAGTCAGTCA	CTGGCTGG	88	101.9	NM_001003349
*B2M*	CCTTGCTCCTCATCCTCCT	TGGGTGTCGTGAGTACACTTG	CAGCATCC	83	99.5	XM_535458
*HMBS*	GCATGTATGCTGTGGGTCAG	GCATGTCCTGGTCCTTGG	GGCCCTGG	63	101.0	XM_546491
*HPRT*	TGTGCTCAAGGGAGGCTATAA	CCATCACTATTTCTGTTCAGTGCT	ACCTGCTG	75	101.8	NM_001003357
*RPL13A*	CTGCCCCACAAGACCAAG	GGGATCCCATCAAACACCT	CCAGGCTG	65	98.6	ENSCAFG00000003633
*SDHA*	GGTGGCACTTCTACGACACC	ATGTAGTGGATGGCGTCCTG	CTGGCTGG	67	102.5	XM_535807
*TBP*	TCCACAGCCTATCCAGAACA	CTGCTGCTGTTGTCTCTGCT	CTGGAGGA	67	97.4	ENSCAFG00000004119
*YWHAZ*	GTTACTTGGCCGAAGTTGCT	TGCTTGTTGTGACTGATCCAC	TGGTGATG	65	100.7	XM_532287
